# Adefovir dipivoxil induced hypophosphatemic osteomalacia in chronic hepatitis B: a comparative study of Chinese and foreign case series

**DOI:** 10.1186/s40360-018-0212-7

**Published:** 2018-05-16

**Authors:** Nan Chen, Jian-bo Zhang, Qiujie Zhang, Yun-peng Zhao, Li-yan Li, Li-wei Liu, Fei Yu, Xin Yu, Tao Peng, Kuan-xiao Tang

**Affiliations:** 1 Department of Geriatrics, Qilu Hospital of Shandong University, Jinan, Shandong China; 2 Department of Emergency, Qilu Hospital of Shandong University, Jinan, Shandong China; 3 Department of Orthopedics, Qilu Hospital of Shandong University, Jinan, Shandong China; 4 Department of Endocrinology and Metabolism, First People’s Hospital of Jinan City, Jinan, Shandong China; 5 Department of Nephrology, Qilu Hospital of Shandong University, Jinan, Shandong China; 6 Present Address: Department of Geriatrics, Qilu Hospital of Shandong University, No. 107, Wenhua Xi Road, Jinan, Shandong 250012 People’s Republic of China

**Keywords:** Adefovir dipivoxil, Hypophosphatemia, Osteomalacia, Renal insufficiency, Fanconi syndrome

## Abstract

**Background:**

Adefovir dipivoxil (ADV)-induced renal tubular dysfunction and hypophosphatemic osteomalacia (HO) have been given great consideration in the past few years. However, no standard guidance is available due to a lack of powerful evidence from appropriate long-term prospective case-control studies and variations in the definition of renal adverse events. The aim of this study is to clarify clinical features of ADV-related HO in Chinese chronic hepatitis B patients with long-term ADV treatment in Chinese and non-Chinese comparative case series.

**Methods:**

Retrieval of case reports was based on Pubmed, CNKI, Wan Fang and VIP databases using the key words adefovir dipivoxil, hypophosphatemia, osteomalacia and Fanconi syndrome. We divided patients into Chinese (C group) and Foreign (F group) groups according to their nationality. Comparisons involving demographics, clinical manifestations, tests, treatment and prognosis were conducted between the two groups.

**Results:**

Of the patients screened, 120 Chinese patients were identified in the C group, and 32 non-Chinese patients were identified in the F group. The average age of the C group was younger than that of the F group (51.89 years ±10.96 years versus 56.47 years ±11.36 years, *t* = − 2.084, *P* = 0.039). No significant difference was found in gender (male to female, 3.29:1 versus 3:1, *χ*^*2*^ = 0.039, *P* = 0.844). Although there was no significant difference in the duration of ADV therapy before ostalgia onset, the C group tended to develop adverse events earlier, by 2–3 years, while the F group developed adverse events at 4–5 years (*Z* = − 1.517, *P* = 0.129). Prognosis was good after adjustment of the ADV dose and supplemental administration of phosphate and calcitriol. Time to resolution of tubular dysfunction was commenced at the first month, and Chinese patients were more prone to recover in the first 3 months than non-Chinese patients (91.3% of patients in the C group versus 56.3% in the F group, *Z* = − 3.013, *P* = 0.003).

**Conclusions:**

Sufficient attention is required for middle-aged males before and during exposure to long-term ADV therapy, regardless of nationality. The clinical picture, laboratory and radiograph alterations are important clues for those patients and are usually characterized by polyarthralgia, renal tubular dysfunction and mineralization defects. Implementation of an early renal tubular injury index is recommended for patients with higher risk, which would prevent further renal injury.

## Background

Adefovir dipivoxil (ADV) is a nucleotide analogue of adenosine monophosphate, which is effective in viral suppression for both treatment-naive and lamivudine-resistant chronic HBV-infected patients [1]. Chronic hepatitis B virus (HBV) infection affects more than 350 million people worldwide, with 75% living in the Asia-Pacific region [2]. Moreover, chronic HBV infection continues to be a major health problem because it leads to the development of liver cirrhosis and hepatocellular carcinoma and increases the risk of hepatic disease-related death. Various studies have reported that adefovir dipivoxil (ADV) can cause proximal renal tubular complex dysfunction, hypophosphatemic osteomalacia (HO) and even Fanconi syndrome since it was first used in the long-term treatment of chronic hepatitis B in 2002 [3]. However, several pitfalls remain to be explained. First, a unified definition of ADV-related renal dysfunction has not yet been identified. Some studies used serum creatinine or evaluated glomerular filtration rate (eGFR), which mainly reflects glomerular function as an endpoint [4, 5], while some studies only investigated indexes concerning renal tubular absorption [6, 7]. No evidence from case-control studies including Chinese chronic hepatitis B patients have been reported, and sample sizes of previous studies were relatively small [8, 9]. Furthermore, patients not matching the original clinical settings were included. To further determine the clinical features of ADV-induced HO in Chinese chronic hepatitis B patients, we investigated the demographics, clinical spectrum, laboratory tests, treatment and prognosis of ADV-induced HO through a comparison of Chinese and non-Chinese case series.

### Comparative series study

ᅟ

## Methods

### Search strategy

PubMed, Chinese National Knowledge Infrastructure (CNKI), the Chinese literature database (Wanfang) and the Chinese Science and Technology Journal Database (VIP) [10] from October 2002 to October 2015 were searched for case reports and relevant clinical trials. The key words were adefovir dipivoxil, hypophosphatemia, osteomalacia, Fanconi Syndrome, and renal insufficiency. The language was limited to Chinese and English. Search strategies for PubMed are shown below:

#1: Search “adefovir dipivoxil”[All Fields]; #2: Search “Fanconi Syndrome”[Mesh]; #3: Search “osteomalacia”[Mesh]; #4: Search “hypophosphatemia”[All Fields]; #5: Search “renal insufficiency”[All Fields]; #6: Search (#2 OR #3 OR #4 OR #5) AND #1.

Relevant Chinese characters were used as keywords when searching Chinese databases.

All references from articles obtained through the databases were reviewed manually.

Inclusion criteria included three parts as follows: (1) Case reports or clinical studies reporting specific cases and conference abstracts with no formal published articles. (2) Safety-related events restricted to hypophosphatemia, osteomalacia and Fanconi syndrome. (3) Events listed occurred during the use of ADV in mono-therapy or combination therapy in naïve or rescue treatment for HBV infection.

Exclusion criteria included the following: (1) Published data that were recorded repeatedly or were lacking in details (e.g., without exact values for hypophosphatemia). (2) Clinical articles without specific case reports or including patients co-infected with HIV or hepatitis C. (3) Meta-analyses, systemic reviews and conference speeches with lack of details.

### Data selection

Two authors (Nan C and Jian-bo Z) independently screened the titles and abstracts of the collected citations from primary searches. Relevant clinical studies were downloaded for specific case reports, as well as those meeting inclusion criteria. Disagreements between the two authors were resolved by discussion and, if needed, arbitrated by a third author (Kuan-xiao T).

### Data extraction

**Fig. 1:**
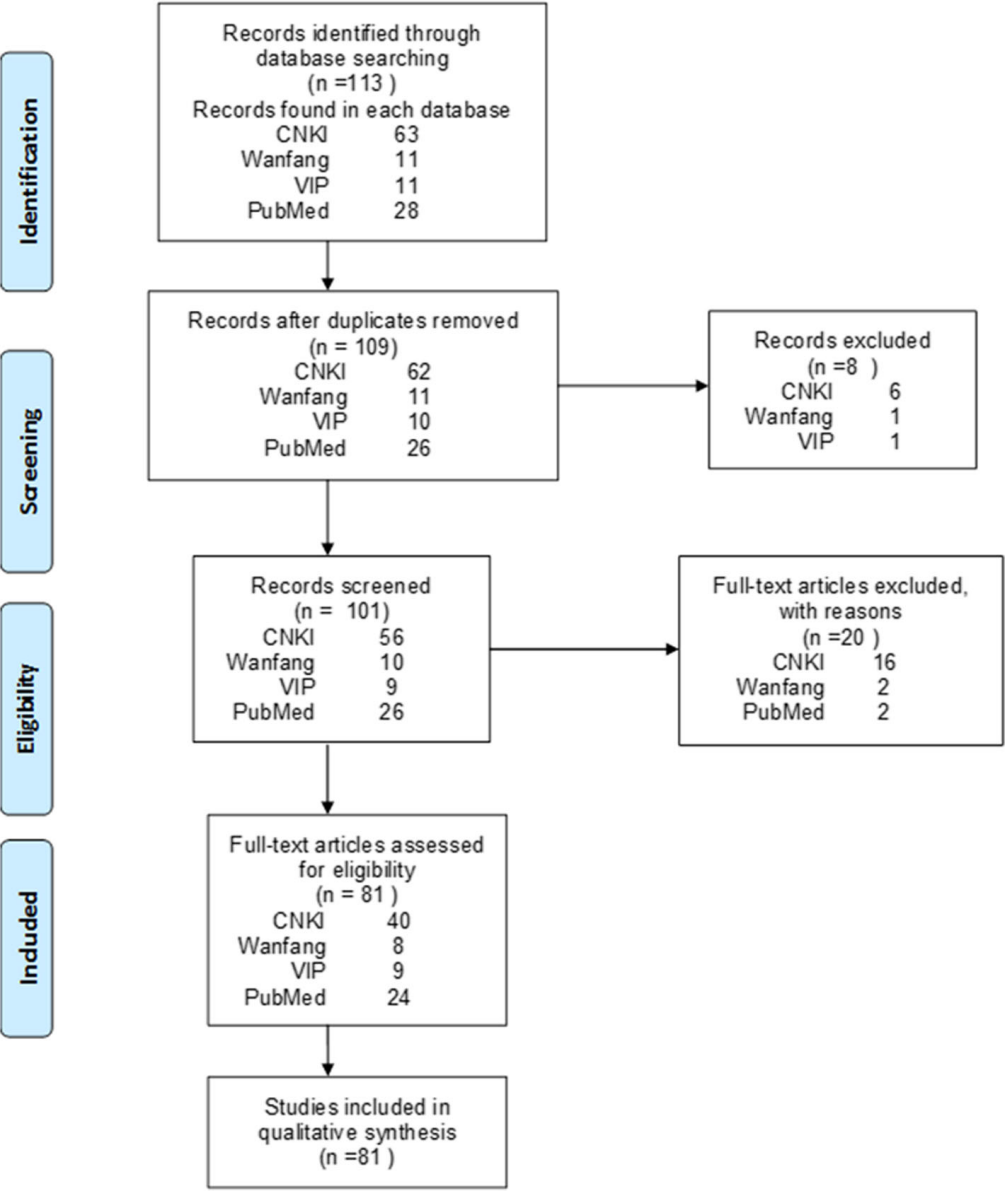
Flow diagram for study selection

A flow chart of the study selection process and exclusion criteria is shown in Fig. 1. One hundred and thirteen records were identified by the electronic database search. After removing duplicates, 109 articles were screened according to the inclusion/exclusion criteria. The remaining 101 full-text articles were assessed for eligibility. Of these articles, 20 were excluded (see S2 File for a list of reasons for exclusion). The remaining 81 articles were included in the final review. We divided patients into Chinese and Foreign groups according to their nationality (C for the Chinese group and F for the Foreign group). Data such as demographics, history (duration of CHB, hepatitis-related complications and comorbidity) and drug details (antiviral drugs, dose, time of starting and maintaining), clinical manifestation, tests, treatment and prognosis were obtained from published sources. The index of laboratory tests was rated according to the National Cancer Institute Common Toxicity Evaluation Standard (NCICTC) Edition 2.0 [11]. For hypophosphatemia, Grade 1 was defined as 0.60- < 0.80 mmol/L, Grade 2 was defined as 0.30- < 0.60 mmol/L, and Grade 3 was defined as < 0.30 mmol/L. For increased serum creatinine, Grade 1 was defined as ≤132.6 μmol/L (≤1.5 mg/dL), Grade 2 was defined as 132.6 μmol/L- ≤ 176.8 μmol/L (1.5- ≤ 2.0 mg/dL), and Grade 3 was defined as 176.8 μmol/L - ≤ 265.2 μmol/L (2.0- ≤ 3.0 mg/dL). The grade classifications are well accepted [12].

### Statistical analysis

Normally distributed data were described as the mean ± standard deviation, while a non-normal distribution was described as the median (inter-quartile range). Two independent sample *t*-tests were used for normally distributed data, while non-parametric tests were used for non-normal data. The count data were analyzed by a *Chi-square* test or *Fisher’s exact* test. A *Mann-Whitney U* test of the two independent samples was used in count data with index variables distributed in one-way orderly, while a *Kruskal-Wallis H* test and rank correlation analysis were used for ordinal categories with different properties. A two-tailed *P-*value < 0.05 was considered to be statistically significant.

## Results

### Demographics (see Table 1)

There were 152 patients eligible for the study, with 120 patients allocated to the C group and 32 non-Chinese patients allocated to the F group. The C group was significantly younger than the F group (51.89 ± 10.96 years versus 56.47 ± 11.36 years, *t* = − 2.084, *P* = 0.039). Of all the patients in both groups, the age distribution illustrated a gender difference, with males being predominant in the middle-aged group (62/116, 53.4%) and females being the majority in the older group (18/36, 50%) (*Z* = − 3.640, *P* < 0.001). No similar tendency was detected in between-group comparisons, and there was no significant difference between the groups regarding gender (male to female, 3.29:1 versus 3:1, *χ*^*2*^ = 0.039, *P* = 0.844) and gender proportion among different age groups (males represented 57.6% versus 37.5% (*Z* = − 0.831, *P* = 0.406), females represented 46.4% versus 62.5% (*Z* = − 0.956, *P* = 0.339)). Asian nationalities accounted for 84.4% in the F group, which were mostly Korean (40.6%) and Japanese (31.3%). The other patients included 2 from France, 2 from Italy and 1 from Spain.

**Table 1:** Baseline characteristics in the C and F group

Characteristics	Descriptive statistics	C group	F group	Df	*P* Value
Case number	*n*	120	32		
Age	Mean ± SD	51.89 ± 10.96	56.47 ± 11.36	150	0.039^a^
	Range	22–79	31–81		
Gender				1	0.844^c^
Male	*n* (%)	92 (76.7%)	24 (75.0%)		
Female	*n* (%)	28 (23.3%)	8 (25.0%)		
Period of pain (months)	*n*	86	17	–	0.098^b^
	Median (Interquartile range)	18 (12–26.5)	13 (5.5–24)		
	Range	1–102	1–72		
ADV total treatment time (months)	*n*	115	30	–	0.939^b^
	Median (Interquartile range)	60 (36–72)	57 (38–69)		
	Range	5–144	9–132		
ADV treatment time of pain onset (months)	*n*	87	22	–	0.153^b^
	Median (Interquartile range)	36 (24–48)	47.5 (28–60)		
	Range	2–95	1–90		
BMI (kg/m^2^)	*n*	8	5	11	0.192^a^
	Mean ± SD	21.21 ± 1.85	23.44 ± 3.98		
	Range	19.53–24.70	18.00–28.00		
Initial time of pain relief (months)	*n*	76	20	–	0.003^b^
	Median (Interquartile range)	1.5 (1–2)	3 (2–6)		
	Range	0.3–6.0	0.5–18.0		
Initial time of serum Phos. Rising (months)	*n*	53	11	–	0.115^b^
	Median (Interquartile range)	2 (1–3)	3.5 (1.75–6)		
	Range	0.3–12.0	0.5–11.0		

*ADV* Adefovir Dipivoxil, *BMI* body mass index, *Phos.* phosphate

^a^Two independent sample *t* test

^b^Two independent sample *Mann-Whitney U* test

^c^ *Fisher’s exact test*

### History

#### ADV history

The average duration of ADV therapy was 60 (36–72) months in C group and 57 (38–69) months in the F group (*Z* = − 0.076, *P* = 0.939), while the duration of ADV therapy before ostalgia onset was 36 (24–48) months versus 47.5 (28–60) months (*Z* = − 1.428, *P* = 0.153). Two to five years of ADV therapy accounted for most patients in both groups (55.7% versus 63.3%, *Z* = − 0.346, *P* = 0.729). Although the differences were not significant, the C group tended to develop ostalgia 1–2 years earlier than the F group (*Z* = − 1.517, *P* = 0.129). The ADV dosage was administered at 10 mg daily, except for 3 patients given 20 mg daily and 1 patient given 15 mg daily in the C group.

#### Other history

The median duration of CHB was 120 (84–195) months in the C group and 132 (46.5–210) months in the F group (only 5 patients recorded). No significant difference was observed in the proportion of liver cirrhosis (95.8% in the C group versus 83.3% in the F group, *P* = 0.253), LAM therapy before exposure to ADV (61.29% versus 76.50%, χ^2^ = 1.739, *P* = 0.187) or LAM add-on with ADV (38.71% versus 23.50%, χ^2^ = 0.849, *P* = 0.357). In addition, there were 6 different comorbid diseases in the C group, including 11 patients with hypertension, 7 with diabetes, and one each with gout, chronic bronchitis, Turner’s syndrome and atopic dermatitis. In the F group, no available details were recorded except that 1 patient had epilepsy and 4 underwent surgery (femoral surgery in 2, kidney transplantation in 1 and subtotal gastrectomy in 1).

### Clinical manifestation

#### Symptoms

All patients presented with persistent ostalgia, occurring originally in the lower limbs (e.g., ankle, knee) and aggravated after load-bearing. Commonly, pain perception progressed gradually from the lower limbs to the thorax, ribs, and upper joints, leading to difficulty in motion, staggering gait and passive position. Moreover, the patients usually complained of fatigue and loss of appetite. Some of them could barely stand up and pursue daily activities. Additionally, it is noted that 14 patients in the C group had non-specific symptoms, such as nocturia and peripheral paresthesia, before the initial onset of ostalgia.

### Physical examination

**Fig. 2:**
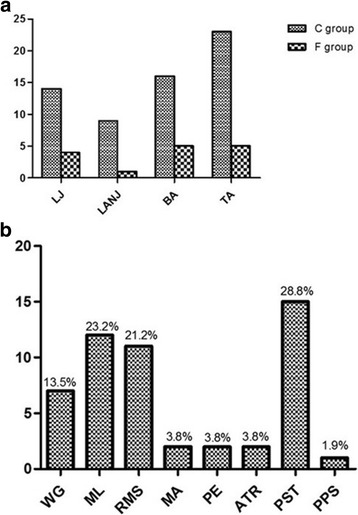
Tenderness distribution in the C and F groups and abnormal signs in the C group. **a** Thirty-three and 8 patients had tenderness in the C and F group, respectively. No significant difference was detected in the LJ (*P* = 1.000),LANJ (*P* = 0.653), BA (*P* = 0.702) and TA (*P* = 0.663) of the two groups. A *Fisher’s* exact test was used in statistical analysis. LJ: lower joint. LANJ: lower limb area of non-joint. BA: back area. TA: thoracic area. **b** The first four abnormal signs recorded in the C group were PST (28.8%), ML (23.1%), RMS (21.2%), and WG (13.5%). WG: waddling gait. ML: mobility limitation. RMS: reduced muscle strength. MA: muscle atrophy. PE: paresthesia. ATR: abnormal tendon reflex. PST: positive orthopedic special test. PS: pathological sign

For all patients, body mass index (BMI) was within the normal range (21.21 ± 1.85 kg/m^2^ in the C group versus 23.44 ± 3.98 kg/m^2^ in the F group, *t* = − 1.389, *P* = 0.192). No significant difference was detected in the tenderness distribution of the two groups (see Fig. 2a). Moreover, Fig. 2b shows the detailed data of the abnormal signs recorded in the C group, which showed that a positive orthopedic special test (PST) had the highest incidence, followed by mobility limitation (ML), reduced muscle strength (RMS), and waddling gait.

### Examinations

#### Laboratory examination (Table 2)

##### Bone mineral metabolism

No significant difference was found in the reduction of serum phosphorus between the two groups. A Grade 1 decrease (0.60- < 0.80 mmol/L) was recorded in a total of 32.7% in the C group versus 25.8% in the F group, a Grade 2 (0.30- < 0.60 mmol/L) decrease was recorded in 63.7% versus 67.7%, and a Grade 3 (< 0.30 mmol/L) decrease was recorded in 3.5% versus 6.5% (*Z* = − 0.880, *P* = 0.379). In addition, 71.4% versus 81.5% of patients had normal excretion of urine phosphorus (*Z* = − 0.617, *P* = 0.657). Moreover, 71.6% versus 63.2% of patients developed hypocalcemia (*Z* = − 0.720, *P* = 0.472). Interestingly, 85.7% versus 88.9 and 78.8% versus 83.3% of patients had a normal level of 25-(OH) D3 (*Z* = − 0.249, *P* = 0.804) [13–17] and PTH (*Z* = − 0.289, *P* = 0.772), respectively. The C group had more information regarding bone biochemical metabolism, and 5.3, 93.8 and 76.9% of patients had increased levels of osteocalcin, Type I tropocollagen amino terminal extension of the peptide (PINP), and special series I type collagen peptide carboxyl end β (β-CTX), respectively, which reflected activation of both osteoblasts and osteoclasts. Though the F group had a higher mean value of alkaline phosphatase (AKP), 81.8% versus 100% of patients had drastically increased AKP. It is not difficult to conclude that patients with ADV-induced HO have hypophosphatemia, hypocalcemia, and increased levels of alkaline phosphatase (AKP), which is essentially different from osteoporosis in bone and mineral metabolism, with the latter including over-activated bone reabsorption. Interestingly, most patients had a normal level of 25(OH)VitD3 in both groups.

**Table 2:** Biochemical parameters in the C and F group

Biochemical parameters^*^		C group		F group	Df	*P* value	Ref. range
	*n*	mean±SD or median (IQ range)	*n*	mean±SD or median (IQ range)			
Blood							
PH^#^	37	7.36(7.33–7.39)	4	7.32(7.28–7.36)	–	0.109^b^	7.35–7.45
Phos. (mmol/L)	117	0.54 ± 0.15	31	0.49 ± 0.14	146	0.115^a^	0.81–1.45
Ca^2+^ (mmol/L)	81	2.18 ± 0.13	19	2.11 ± 0.28	98	0.305^a^	2.25–2.75
K^+^ (mmol/L)	80	3.48 ± 0.40	10	3.75 ± 0.42	88	0.048^a^	3.5–5.5
UA (umol/L)	65	110(90.65–131.85)	13	107(95.20–152.85)	–	0.406^b^	210–420
BG (mmol/L)	33	5.02(4.75–5.36)	4	5.55(4.70–5.93)	–	0.352^b^	3.9–5.6
SCr (umol/L)	69	107.71 ± 28.75	19	115.65 ± 32.20	86	0.302^a^	44.2–88.4
25-(OH)VitD3 (ng/ml) ^&^	42	15.45(12.90–21.75)	9	23.50(11.80–29.80)	–	0.236^b^	9–52
PTH (ng/ml)	52	30.56(23.84–48.22)	18	28.37(20.00–46.96)	–	0.657^b^	15–65
AKP (IU/L)	94	240.5(179.8–317.0)	25	698(324.5–1160.0)	–	<0.001^b^	40–150
Urine							
Phos./24 h (mmol/24 h)	35	18.81 ± 10.39	8	21.69 ± 8.98	41	0.473^a^	12.9–42.0
Ca^2+^ /24 h (mmol/24 h)	26	9.23 ± 4.31	9	7.19 ± 4.50	33	0.234^a^	2.5–7.5
Protein/24 h (mmol/24 h)	34	1.00 ± 0.49	10	1.16 ± 0.43	42	0.361^a^	< 0.15
NAG (U/L)	10	35.5 (27.0–55.15)	4	22.8 (13.65–43.35)	–	0.239^b^	2–18
β2-MG (mg/L)	14	13.71 (5.13–81.3)	4	77.1 (50.58–119.32)	–	0.110^b^	< 0.2

*Different sample size has been given for different parameters in the C and the F group order

^#^Only 3 patients had urine PH record, they were 6.5, 7.5, 7.5, respectively

^a^Two independent sample *t* test

^b^Two independent sample *Mann-Whitney U* test

^**&**^The range was set according to published reports [13–17]

##### Biochemical metabolism

No significant difference was found in the level blood uric acid, serum creatinine, plasma glucose and serum PH. Hypouricemia was seen in 90.8% of patients in the C group and 100% in the F group (*Z* = − 1.089, *P* = 0.276). The C group was more prone to experiencing hypokalemia, with an average serum potassium of 3.48 ± 0.40 mmol/L versus 3.75 ± 0.42 mmol/L (*t* = − 2.01, *P* = 0.048). Of note, 88.4% versus 78.9% of patients had increased serum creatinine in Grade 1 (*Z* = − 1.090, *P* = 0.276). Interestingly, positive urine glucose was detected in both groups, even in those without diabetes. Both groups had markedly elevated urinary β2-microglobulin (β2-MG). Though no significant differences were noted, the C group had a lower rate of N-acetyl beta D glucose anhydride enzyme (NAG) increase (87.5% versus 100%) and proteinuria over 0.5 g per 24 h (82.4% versus 100%). Furthermore, the C group also had less tendency to develop acidosis despite a median serum PH value (40% versus 83.3%) at the lower limit of the normal range.

A significant inverse correlation between age and serum creatinine was found in the C (*γ* = − 0.820, *P* = 0.005) and F group (*γ* = − 0.840, *P* = 0.006). This correlation was not seen in age and serum phosphorus or in serum creatinine and serum phosphorus. For 20 patients with severe proteinuria, no correlation was observed with serum creatinine. Hypophosphatemia, hypouricemia, hypocalcemia, and proximal renal tubular dysfunction were characterized by an increased excretion of urine phosphorus, calcium, β2-MG, NAG, severe proteinuria and renal tubular acidosis, which are commonly present in ADV-related HO. No correlation was found between hypophosphatemia and age or serum creatinine. In contrast to conventional wisdom, serum creatinine did not show a positive trend with age and was insensitive for the detection of early pathological alterations.

### Imaging examination

No significant difference was found in the results of bone mineral density by dual energy X-ray absorptiometry (DEXA), with 65.2% in the C group versus 62.5% in the F group, which had a T score below − 2.5 standard deviation (SD), as well as a distribution of abnormality in the ^99m^Tc-MDP whole body bone scintigraphy. There were increased multiple foci with radiotracer symmetrically distributed in the area of the lower joints (ankles, knees, sacroiliac joints, and hips), upper joints (shoulders), thorax (ribs), back (spines), and skull. In addition, 4 patients in the C group were diagnosed with neurogenic damage through an electromyogram.

### Cytology examination

No comparison was conducted in this situation since there was a significant difference in sample size. Twenty patients in the C group had a renal biopsy, of whom 18 displayed proximal tubular epithelium atrophy and dramatic vacuolization of epithelial cells with a fading of the brush border and 9 had simultaneous glomeruli lesions. The other two patients were diagnosed with IgA nephropathy by electron microscopy. Renal biopsy was reported in only two patients in the F group. One patient had significant renal tubular lesions, while the other had lesions limited to the glomeruli. Three patients had bone biopsies in the F group, and the biopsies mainly showed local osteoid accumulation and a lack of mineralization. One patient had a muscle biopsy in the C and F groups, and no significant abnormality was found in response to myasthenia. Multiple myeloma was excluded for one patient having bone marrow cytology in the C group.

### Misdiagnosis and diagnosis

#### Misdiagnosis

**Fig. 3:**
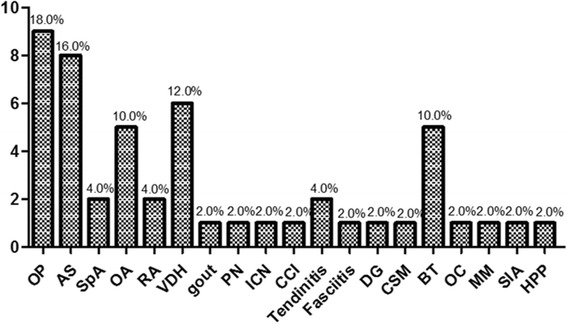
Misdiagnosis in the C group. Osteoporosis accounted for the most common misdiagnosis (18%), followed by AS (16%), VDH (12%), OA (10%) and BT (10%). OP: osteoporosis. AS: ankylosing spondylitis. SpA: spondyloarthropathy. OA: osteoarthropathy. RA: rheumatoid arthritis. VDH: vertebral disc herniation. PN: peripheral neuropathy. ICN: intercostal neuralgia. CCI: costochondritis. DG: degeneration. CSM: cervical spondylosis myelopathy. BT: bone tumor. OC: osteochondroma. MM: multiple myeloma. SIA: sacroiliac arthritis. HPP: hypopotassium periodic paralysis

Twenty-seven patients in the C group (Fig. 3) reported misdiagnosis. Osteoporosis (10/27, 18.0%), ankylosing spondylitis (9/27, 16.0%), lumbar disc herniation (6/27, 12.0%), osteoarthritis (4/27, 10.0%) and bone tumors (4/27, 10.0%) were more frequent. For the F group, only 3 patients had misdiagnoses, including osteoporosis, ankylosing spondylitis and Paget’s disease.

#### Diagnosis

All of the patients were diagnosed with hypophosphatemia and developed hypophosphatemic osteomalacia except for 10 patients in the C group who only reported hypophosphatemia. General damage of proximal renal tubular function, namely, Fanconi syndrome, was recorded in 57 patients in the C group versus 16 in the F group.

### Treatment and prognosis

#### Treatment (Table 3)

##### Antiviral drugs

There was a significant difference in the proportion of ADV dosage adjustment; 89% of patients in the C group versus 67.9% in the F group had treatment discontinuation (*χ*^2^ = 5.652, *P* = 0.017), while 4.4% versus 28.6% had reduced ADV dosage or a prolonged dosage interval (*χ*^2^ = 13.802, *P* < 0.001). Furthermore, 56% versus 36.7% of patients replaced ADV with entecavir after ADV discontinuation (*χ*^2^ = 3.542, *P* = 0.06).

**Table 3:** Treatment in the C and F group*

Treatment	Presence	Absence	Df	*P* value
cADV^#^			1	0.017^b^
C	81(89.0%)	10(11.0%)		
F	19(67.9%)	9(32.1%)		
rADV			1	0.001^b^
C	4(4.4%)	87(95.6%)		
F	8(28.6%)	20(71.4%)		
ETV			1	0.060^a^
C	51(56.0%)	40(44.0%)		
F	10(35.7%)	18(64.3%)		
Phos.			1	0.210^a^
C	53(58.2%)	38(41.8%)		
F	20(71.4%)	8(28.6%)		
Calcium			1	0.001^a^
C	47(51.6%)	44(48.4%)		
F	3 (10.7%)	25(89.3%)		
Calcitriol			1	0.185^a^
C	52(57.1%)	39(42.9%)		
F	12(42.9%)	16(57.1%)		
BM			1	0.755^b^
C	6(6.6%)	85(93.4%)		
F	3(10.7%)	25(89.3%)		

*Ninety-one and 28 patients had treatment in the C and F group, respectively

*cADV* ceased Adefovir Dipivoxil, *rADV* reduced Adefovir Dipivoxil, *ETV* entecavir, *Phos.* Phosphate supplement, *Calcium* Calcium supplement, *Calcitriol* Calcitriol supplement, *BM* drugs regulating bone metabolism

^a^ *Pearson Chi-square test*

^b^Corrected *Pearson Chi-square test*

#### Bone mineral metabolism regulation

There was no significant difference in the proportion of phosphate (58.2% versus 71.4%, *χ*^2^ = 1.570, *P* = 0.210) or vitamin D3 (57.1% versus 42.9%, *χ*^2^ = 1.758, *P* = 0.185) supplementation between the two groups. The C group was more prone to supplement with calcium than the F group (51.6% versus 10.7%, *χ*^2^ = 14.726, *P* < 0.001).

### Prognosis (Table 4)

#### Time of symptom remission

Significant differences were found in the median time of pain remission (1.5 (1, 2) months in the C group versus 3 (2, 6) months in the F group, *Z* = − 2.951, *P* = 0.003), with the C group being more likely to recover in the first 3 months (91.3% of patients in the C group versus 56.3% in the F group, *Z* = − 3.013, *P* = 0.001).

**Table 4:** Time of symptom and serology recovery*

	C group	F group	*P*-value
Initial time of pain relief (months)			0.001^a^
≤1	37(46.3%)	3(18.8%)	
1<T≤3	36(45.0%)	6(37.5%)	
> 3	7(8.8%)	7(43.8%)	
Initial time of serum Phos. Rising (months)			0.099^a^
≤1	19(35.2%)	2(20.0%)	
1<T≤3	25(46.3%)	3(30.0%)	
3<T≤6	6(11.1%)	4(40.0%)	
>6	4(7.4%)	1(10.0%)	

*Eighty and 16 patients recorded the initial time of pain relief in C and F group, respectively, and 54 and 10 patients recorded the initial time of serum Phos. Rising

^a^Two independent sample *Mann-Whitney U* test

#### Recovery of serology

Though no significant difference was observed in the median time of resolution of serum phosphorus (2 (1, 3) months in the C group versus 3.5 (1.75, 6) months in the F group, *Z* = − 1.157, *P* = 0.115), the C group tended to recover in the first three months (81.5% of patients versus 50%, *Z* = − 1.649, *P* = 0.099).

Here, we reported a Chinese woman with generalized bone pain for 8 months without antecedent trauma. The study was approved by the ethics committee of Qilu Hospital of Shandong University, and the study protocol conformed to the ethical guidelines of the 1975 Declaration of Helsinki. The individual in this manuscript had given written informed consent to publish the case details.

**Fig. 4:**
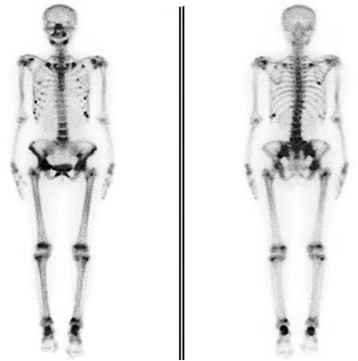
^99m^Tc-MDP diphosphonate bone scintigraphy of the patient discussed above. A technetium bone scan showed multifocal lesions which distributed to the bilateral ankles, knees, hips, pelvis, ribs, and shoulders symmetrically, while the lesions were dispersed in the skull, sternum, spine and limbs

The patient was a 43-year-old woman with chronic hepatitis B for more than eight years. ADV was administered from diagnosis until the occurrence of pain initiating from the foot joints eight months ago. No remarkable history of hypertension or renal insufficiency was noted. She experienced progressive bone pain extending from the lower limbs to the lower back and ribs in the next two months. Laboratory tests showed a serum phosphorus level of 0.62 mmol/L, a calcium level of 2.21 mmol/L, and an AKP of 160 U/L. Urine testing revealed proteinuria and glucosuria. Electrolytes from the urinalysis showed a calcium level of 19.84 mmol/24 h, a potassium level of 74.48 mmol/24 h, and a phosphate level of 35.77 mmol/24 h. Impaired renal tubular reabsorption was noted as a drastic increase of urine micro-albumin (75.8 mg/L) and β2-microglobulin (11.8 mg/L). Bone biochemical metabolism tests showed a decreased level of 25-OH vitamin D with no further abnormalities. DEXA detected a decreased femur neck mineral density (BMD) with a mean Z-score of − 3.0 standard deviations. The whole-body ^99m^Tc-MDP diphosphonate bone scintigraphy (Fig. 4) showed increased symmetrical uptake in the ankles, knees, hips, sacroiliac joints and multiple ribs and increased diffuse uptake in the limbs, pelvis, spine, sternum and skull. A clinical diagnosis of ADV-induced HO with secondary Fanconi syndrome was suspected. ADV was discontinued and supplementation with oral phosphate, calcium, and vitamin D was started. The patient’s symptoms improved after two weeks, and the serum phosphorus increased to 0.63 mmol/L. Systemic pain was adequately relieved one month after discharge. Two months later, all of the symptoms disappeared, and the serum phosphorus returned to normal (no test data available).

## Discussion

To the best of our knowledge, this is the first study to investigate the differences among the demographics, clinical manifestation, tests, treatment and prognosis in ADV-related HO between Chinese and non-Chinese CHB patients. Several studies have shown that males older than 50 years are at an increased risk of developing HO [5, 12]. Consistently, middle-aged males were highly vulnerable to HO in our study. Moreover, it seems that being a middle-aged male may be a predictor of HO regardless of nationality. Chinese patients were prone to developing HO earlier, which might due to the difference in sample size. Potential gender-specific association of HO with ADV need to be elucidated in further studies for females. The elderly seemed to be more predisposed to HO. Such considerable age difference in genders regarding the incidence of HO may partly be explained by the variance in bone structure and strength, body fat deposition, sex hormone levels, and the risk of falls between men and women [18]. Intriguingly, the F group is predisposed compared to Asian nationals, probably due to the high incidence of hepatitis B in Asians, race differences and personal genetic susceptibility [19, 20].

Hypophosphatemia mostly occurs one year after ADV therapy in a time- and dosage-dependent manner [12, 21]. As shown in our study, the median course before onset of HO was 3–4 years, with Chinese patients predisposed earlier occurrence, indicating a preexistence of underlying hypophosphatemia. The symptoms of patients varied from mild, such as fatigue, muscle weakness and paresthesia, to severe skeletal pain, disabling myopathy, walking difficulty, and even fractures. However, for some patients, ostalgia never occurred, which might due to differences in duration of CHB, the severity of hypophosphatemia and personal tolerance. Non-specific malnutrition causing muscle atrophy is commonly present in CHB patients with decreased muscle strength, which in turn decreases bone mineral density [22].

Hypophosphatemic osteomalacia (HO) is one of the serious adverse events of ADV, which is mainly related to proximal renal tubular dysfunction. The underlying mechanisms have not been well-explained. ADV is excreted from the kidney in its original form; human organic anion transporter 1 (hOAT1) in the basolateral membrane of the proximal renal tubules [23] and multidrug resistance–associated protein type 2 (MRP2) in the apical membrane [24] control basolateral uptake and luminal excretion, respectively. The polymorphism of genes encoding these two drug transporters may play a role in individual susceptibility [25], leading to generalized dysfunction of proximal renal tubular reabsorption after exposure to ADV. Moreover, ADV has a low-level activity against mitochondrial DNA polymerase-γ when inhibiting HBV-DNA polymerase simultaneously, ultimately leading to mitochondrial dysfunction, impaired oxidative phosphorylation, and tissue injury [26]. Nonetheless, differences in general health and personal tolerance might result in different clinical phenotypes of mitochondrial toxicity [27], leading to differences in the clinical spectrum.

Moreover, it must be outlined that renal abnormalities are frequently observed in patients without any oral antihepatitis B virus treatment. In a cross-section study, Amet et al. [28] found that the prevalence of renal dysfunction in CHB patients was approximately 64.6% and emphasized the necessity for regular monitoring of renal function. Several studies [5, 12, 29, 30] showed middle-aged male patients, especially those with cirrhosis, were more vulnerable, because low-grade renal abnormalities are frequently found in such patients with a history of CHB. Our study showed that over 80% of patients in both groups developed renal tubular dysfunction with elevated β2-MG and NAG. Furthermore, severe proteinuria frequently occurred in the patients, even if serum creatinine was still normal or near normal. The underlying mechanisms are unknown. One study reported that the expression of hepatitis B virus in kidney tissues through an immune-complex mechanism [31] results in direct pathological lesion and chronic immunologic injury. Interestingly, in contrast to common sense, serum creatinine is not positively correlated with age in such patients. However, it should be noted that the extent to which ADV contributed to the observed renal abnormalities was confounded by the concomitant use of nephrotoxic medications, including valproic acid in epilepsy [32], and co-morbid conditions such as diabetes, hypertension and non-alcoholic fatty disease [33].

A small number of patients in both groups were exposed to LAM therapy before ADV or had ADV treatment after LAM intolerance. Whether LAM increases renal toxicity remained undetermined until recently. Recent studies showed that LAM was not a risk factor for renal impairment in either the univariate or multivariate analysis [5]. Additional studies are necessary to elucidate the renal safety of LAM.

Metabolic disturbances of bone are common complications in patients with chronic liver disease, mainly osteoporosis [34]. Our study showed that more than 80% of patients had cirrhosis, over 70% had osteopenia, and over 60% had moderate hypophosphatemia. Though osteoporosis accounted for a large portion of misdiagnoses, a total of 142 (93.4%) patients were diagnosed with osteomalacia. Multiple factors, such as vitamin D deficiency, low levels of osteocalcin, PTH, insulin growth factor-1(IGF-1), hypogonadism, behavioral factors such as low body mass index (BMI), malnutrition [35], and a sedentary lifestyle with physical inactivity [36] are frequently observed in these patients, especially those with cirrhosis. There is no doubt that long-term administration of antiviral therapy in such patients would further increase the risk of bone loss resulting from decreased remodeling of bone in the absence of adequate phosphorus and calcium, leading to softening and distortion of the skeleton. Unexpectedly, over 85% of patients maintained a normal level of 25-(OH) D3 in our study [37], which may be explained by the heterogeneity of the subjects in different reports and by differences in the statistical methods used.

According to our present study, routine renal function tests based on serum creatinine are not sensitive enough to detect early abnormalities of renal structure and function [38]. Previous studies have demonstrated biomarkers such as fractional excretion of filtered phosphate (FEPO4) [39], retinol-binding protein (RBP) and N-acetylglucosamine (NAG) [40] may be used for early renal tubular injury detection during long-term ADV treatment. No complete data can be found in the application of the above indices in our study, which implicates that tests reflecting early kidney injury are still not yet widely implemented in the daily clinical setting. In addition, significant differences found in serum potassium and AKP may due to differences in the sample size and methods used. ADV-related nephrotoxicity manifests as a proximal renal tubular dysfunction and is primarily featured by the onset of gradual decreases in serum phosphorus, usually mild to moderate in severity, and can be accompanied by changes in serum potassium, bicarbonate, uric acid, glycosuria, and proteinuria. Though lacking predictor analysis, we still emphasize the importance of monitoring serum phosphorus, calcium, AKP, urine electrolytes, serum uric acid, urine routine testing, and biomarkers of renal tubular injury (NAG, β2-MG, FEPO4) during long-term ADV therapy. Special care is needed, especially for differential diagnoses, since oeteomalacia and osteoporosis commonly co-exist in such patients, and both are characterized by reduced bone mineral density, diffuse bone pain and susceptibility to fracture. Radiologically, pseudofractures are seen most commonly in stress-bearing bones of osteomalacia patients in 99mTc-MDP whole body bone scintigraphy. 99mTc-MDP whole body bone scintigraphy would be preferable to diagnose osteomalacia primarily characterized by mineralization defects, despite the ability of DEXA to diagnose osteoporosis.

Treatment of ADV-induced HO is still not well established. Modification of risk factors involving smoking, alcohol and malnutrition is of prime importance. Some studies suggest that changes of the antiviral drug are more favorable than a phosphate regimen for the purpose of bone protection, preferring dosing-interval adjustment [41, 42]. In this respect, our study showed that Chinese patients were more prone to ADV withdrawal, while non-Chinese patients were prone to dosage adjustment. However, comprehensive consideration, including the potential risk for viral breakthrough, aggravation of liver function, therapy cost and tolerance, is essential before adjustment. For most mildly affected patients, dosage interval adjustment or termination is sufficient without further phosphate supplementation, while for severe cases, phosphate supplementation is indispensable. Our study indicated that both groups adopted phosphate supplementation, in combination with vitamin D3 supplementation, whereas Chinese patients more commonly had calcium supplementation. Several patients were treated with drugs that regulate bone metabolism, such as calcitonin. It is important to note that calcitonin is not optimal, since it can reduce renal tubular phosphate reabsorption and inhibit osteoclast activity even though it has obvious analgesic functions [43]. Other drugs affecting proximal renal tubular reabsorption, such as valproic [32], should also be adjusted. Individual differences in bone remodeling period, duration of CHB, and comorbidity (e.g., osteoporosis) may result in different times to remission. Our study showed that the resolution of proximal renal tubular dysfunction started in the first month, and most patients did not resolve completely until three months of treatment, with Chinese patients being prone to resolve earlier. We consider such variable responses to therapy to be a result of race differences and bias in reports. On the whole, ADV-induced abnormalities are reversible after appropriate drug modification, and remission is predictable at the first month of therapy.

In addition, differences in group samples and the absence of multivariable analysis limit the power of our study. A large clinical prospective case-control study is required to further elucidate the risk factors and assist clinical implementation.

There are limitations in this study. First, this is a retrospective study, and it was hard to collect all of the required lab parameters. Second, multivariate analysis is a useful way to show figures more clearly, but considering the loss of effective information, it is impossible to analyze in that way. Third, the smaller population in the F group compared to the Chinese group leads to a deviation error in the conclusions. Further investigation with more detailed information is in process and will be published in the future.

To date, there are no common definition for adequate vitamin D status measured as 25(OH)D serum concentrations, and the range of 25-(OH) D3 varies from article to article. National Institute of Health, Office of Dietary Supplements, considered 25(OH) D levels < 12 ng/ml as 25-(OH) D3 deficiency, and 12- < 20 ng/ml as insufficient and > 20 ng/ml sufficient. However, some researchers and clinicians defined 25-(OH) D3 deficiency as < 20 ng/ml [44]. Besides, explanations of the normal ranges for 25-(OH) D3 in adults and children from ARUP laboratories (one of the major reference laboratories in the USA) are present in http://ltd.aruplab.com/Tests/Pdf/203. In the current study, we collected the information from various case reports and other clinical reports, which is closed to NIH Office of Dietary supplements definition. Moreover, the level of 25-(OH) D3 is a composition of endogenous synthesis of 25-(OH) D3 as well as absorption of exogenous 25-(OH) D3, and variation between different races and even different countries because of diet, geological and other factors might also contribute to the difference for the normal range of 25-(OH) D3.

In conclusion, middle-aged male patients have a higher risk of HO during long-term ADV treatment, especially those with cirrhosis, and Chinese patients are more prone to develop HO earlier than foreign patients. Sufficient attention is needed for older females, regardless of nationality. The clinical picture and laboratory and radiograph alterations are the most important factors in the diagnosis for those suspected of HO and are characterized by polyarthralgia, renal tubular dysfunction, and mineralization defects. Routine tests are not appropriate. Monitoring early indicators reflecting proximal renal tubular function before and during exposure to ADV is paramount, because renal abnormalities are still revisable after prophylactic or therapeutic intervention. Prognosis is good after adjustment of ADV therapy, and phosphate as well as calcium supplementation might be necessary otherwise.

## Conclusions

Our results show that attention should be paid to middle-aged males before and during exposure to long-term ADV therapy in both the Chinese patients and selected non-Chinese patients included in our study. Clues such as clinical picture and laboratory and radiograph alterations are necessary for suspected patients, and they are characterized by polyarthralgia, renal tubular dysfunction and mineralization defects. Implementation of an early renal tubular injury index is beneficial for higher-risk patients to avoid further renal injuries.

## Abbreviations

ADV: Adefovir dipivoxil
AKP: Alkaline phosphatase
BMI: Body mass index
CHB: Chronic viral hepatitis B
CNKI: Chinese National Knowledge Infrastructure
DEXA: Dual energy X-ray absorptiometry
eGFR: Evaluated glomerular filtration rate
HO: Hypophosphatemic osteomalacia
IGF-1: Insulin growth factor-1
LAM: Lipoarabinomannan
ML: Mobility limitation
MRP2: Multidrug resistance–associated protein type 2
NAG: N-acetyl beta D glucose anhydride enzyme
NAG: N-acetylglucosamine
PST: Positive orthopedic special test
RBP: Retinol-binding protein
RMS: Reduced muscle strength
SD: Standard deviation
VIP: Chinese Science and Technology Journal Database
β-CTX: Collagen peptide carboxyl endβ
